# Evaluation of Antimicrobial Interventions against *E. coli* O157:H7 on the Surface of Raw Beef to Reduce Bacterial Translocation during Blade Tenderization

**DOI:** 10.3390/foods8020080

**Published:** 2019-02-20

**Authors:** Peter M. Muriana, Jackie Eager, Brent Wellings, Brad Morgan, Jacob Nelson, Kalpana Kushwaha

**Affiliations:** 1 Robert M. Kerr Food & Agricultural Products Center, Oklahoma State University, Stillwater, OK 74078-6055, USA; jacob.nelson@okstate.edu (J.N.); kalpana.kushwaha@gmail.com (K.K.); 2 Department of Animal and Food Sciences, Oklahoma State University, Stillwater, OK 74078-6055, USA; eager.jackie@gmail.com (J.E.); brentwellings@yahoo.com (B.W.); 3 Performance Food Group, 2205 Tanglewood Circle, Stillwater, OK 74074, USA; brad.morgan@pfgc.com

**Keywords:** *E. coli* O157:H7, non-intact beef, mechanical tenderization, blade tenderization, antimicrobial interventions, translocation

## Abstract

The US Department of Agriculture, Food Safety Inspection Service (USDA-FSIS) considers mechanically-tenderized beef as “non-intact” and a food safety concern because of the potential for translocation of surface *Escherichia coli* O157:H7 into the interior of the meat that may be cooked “rare or medium-rare” and consumed. We evaluated 14 potential spray interventions on *E. coli* O157:H7-inoculated lean beef wafers (~10^6^ CFU/cm^2^, *n* = 896) passing through a spray system (18 s dwell time, ~40 pounds per square inch, PSI) integrated into the front end of a Ross TC-700MC tenderizer. Inoculated and processed beef wafers were stomached with D/E neutralizing broth and plated immediately, or were held in refrigerated storage for 1-, 7-, or 14-days prior to microbial enumeration. Seven antimicrobials that showed better performance in preliminary screening on beef wafers were selected for further testing on beef subprimals in conjunction with blade tenderization. Boneless top sirloin beef subprimals were inoculated at ~2 × 10^4^ CFU/cm^2^ with a four-strain cocktail of *E. coli* O157:H7 and passed once, lean side up, through an integrated spray system and blade tenderizer. Core samples obtained from each subprimal were examined for the presence/absence of *E. coli* O157:H7. The absence of *E. coli* O157:H7 in core samples correlated with the ability of the antimicrobials to reduce bacterial levels on the surface of beef prior to blade tenderization.

## 1. Introduction

Beef is a desirable food staple in the United States and consumer expectations for product quality, consistency, and tenderness have always been a high priority by the US beef industry [1]. In complying with these priorities, numerous approaches have been implemented in order to make beef a more palatable and enjoyable consumer experience. These include methods to provide proper feed rations such as grain-fed cattle that produce better marbling than grass-fed cattle [2]. However, during the grain shortage of 1974, the number of grass fed cattle increased and researchers turned to mechanical methods of tenderization to enhance the palatability of finished beef products. Studies have shown that mechanical tenderization does result in the improvement of meat tenderness [3,4,5,6,7] and it has been estimated that over 90% of hotel, restaurant, and institutional operations use beef that has been blade tenderized [8].

Safety concerns for *E. coli* O157:H7, a major Shiga toxigenic *E. coli* (STEC) serotype associated with raw beef products, have been heightened since a major outbreak in 1994 resulting in 732 illnesses and four deaths from the consumption of insufficiently cooked ground beef, a ‘non-intact’ beef product [9]. The US Department of Agriculture, Food Safety Inspection Service (USDA-FSIS) then declared *E. coli* O157:H7 an “adulterant” in raw ground beef and on beef carcasses [10]. In 2011, the USDA-FSIS proposed six additional “non-O157” STEC serotypes as adulterants in beef (O26, O45, O103, O111, O121, and O145) for which testing was implemented in 2012 [11]. According to the USDA-FSIS, “intact” beef is a “cut of whole muscle(s) that has not been injected, mechanically tenderized or reconstructed” [12] and therefore mechanically tenderized beef products have been placed in a category considered to be “non-intact” beef. One of the first concerns with the safety of non-intact beef was brought forth from research performed at Kansas State University [13] whereby they identified that blade tenderization of *E. coli* O157:H7 surface-inoculated beef steaks could lead to the internalization of 3–4% of the surface bacteria (i.e., translocation) and that cooking to lower internal temperatures may not sufficiently eliminate the translocated bacteria. In 2001, the issue of potential risk from mechanically-tenderized beef products was brought to the attention of USDA-FSIS and the National Advisory Committee for the Microbiological Criteria for Foods (NACMCF) recommended further studies on this topic [12]. Since then, there have been several outbreaks of *E. coli* O157:H7 attributed to mechanically-tenderized beef [14,15] and USDA-FSIS has been besieged by consumer interest groups demanding that mechanically-tenderized beef products should be labeled as such because “consumers deserve to know if the beef they are eating has been processed this way”. In spite of resistance from the beef industry, the USDA-FSIS has recently issued a final rule on the labeling of mechanically tenderized beef, whereby the name “mechanically-tenderized” must appear as part of the name of the beef product [16]. Studies have been conducted to examine various issues in regard to mechanical tenderization, including the “translocation” of *E. coli* O157:H7 to the interior meat when subject to mechanical tenderization [17], survival of *E. coli* in tenderized beef [18], and what happens to translocated O157 and non-O157 STEC when such steaks are subjected to heating on a grill [19,20].

This study focuses on the use of an integrated tenderizer-spray system to examine various antimicrobials to inhibit *E. coli* O157:H7 on the surface of lean beef wafers to mimic the surface of intact beef prior to blade tenderization. The better performing antimicrobials were then used again in actual blade tenderization experiments to be compared with data recovered from beef core samples after blade tenderization to determine if the translocation to beef interiors can be reduced by antimicrobial spray interventions. Validated interventions using antimicrobials that are proven to reduce the surface incidence of *E. coli* O157:H7 and other STEC may mitigate health risks from internalized pathogens due to mechanical tenderization.

## 2. Materials and Methods

### 2.1. Bacterial Strains

A four strain “cocktail” of *E. coli* O157:H7 (ATCC 43890, ATCC 43894, ATCC 43895, ATCC 35150) was used. These strains were outbreak isolates associated with beef and several (ATCC 43894, ATCC 43895, and ATCC 35150) are considered “acid resistant”, and therefore present a good challenge against acidic antimicrobial treatments [21]. Variants of these strains were recovered that were resistant to gentamycin (10 μg/mL; Sigma-Aldrich, St. Louis, MO, USA) and rifamycin SV (10 μg/mL; MP Biomedicals LLC., Solon, OH, USA) by passage on media containing these antibiotics (in succession). The plating of constitutively resistant strains on non-harsh media containing the antibiotics allows their selective recovery from indigenous background flora on non-sterile meat samples. No adverse cross-reactions were observed from cross-streaks of individual strains on Difco^TM^ tryptic soy agar media (TSA; Becton-Dickinson & Company, Sparks, MD, USA). Stock strains were cultured separately in Difco^TM^ tryptic soy broth (TSB) containing 1% glucose at 37 °C for 18–20 h. The strains grew to near-identical levels in individual culture and were mixed in equal proportions to obtain a mixed-strain cocktail in a 50 mL centrifuge tube, and diluted to an appropriate use level in 0.1% buffered peptone water (PBW) to achieve the desired CFU/cm^2^ using 0.1 mL inoculum in a 2-in diameter circle (lean beef wafers or beef subprimals).

### 2.2. Biosafety Level 2 (BSL-2) Laboratory Precautions

**Figure 1:**
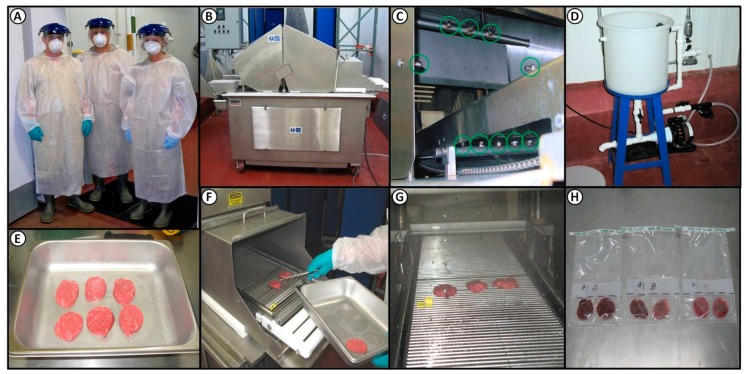
Phase 1, evaluating antimicrobial efficacy against *E. coli* O157:H7-inoculated lean beef discs. (**a**) BSL-2 lab personal protection equipment; (**b**) Ross blade tenderizer; (**c**) 10 spray nozzles (circled) integrated 360° into the entrance of the blade tenderizer; (**d**) solution reservoir and pump system in an adjacent room; (**e**) inoculated lean beef discs; (**f**) placement of inoculated discs on conveyor belt; (**g**) inoculated discs travelling through the 360° spray zone for 18 s dwell time; (**h**) processed beef discs are sampled in pairs prior to processing for microbial plating or vacuum-packaged for later analysis.

These experiments took place in a Biosafety Level 2 (BSL-2) pathogen processing facility in Oklahoma State University’s Robert M. Kerr Food and Ag Products Center (Stillwater, OK, USA). Protocols for experiments, pathogen use, personnel protection, and post-process sanitation were examined and approved by an institutional biosafety committee (IBC) within the university compliance office and shared with USDA-FSIS prior to implementation. Individuals were required to wear adequate personnel protection equipment (PPE) including work overalls, boots, a long-sleeve boot-length disposable Tyvec outer garment, gloves, a surgical mask, and a face splash shield (Figure 1). The facility room where wet processing occurred using *E. coli* O157:H7 was sanitized afterwards by hot water spray, followed by hypochlorite spray, and the room air was then saturated with quaternary ammonium chloride (Bi-Quat) fog misted into the air by a pressurized canister (Birko Corp., Henderson, CO, USA). Select areas in the work area were then swab-tested for our specific antibiotic-resistant *E. coli* O157:H7 strains before the room was again available for use.

### 2.3. Processing Lean Beef Wafers and Beef Cores

**Figure 2:**
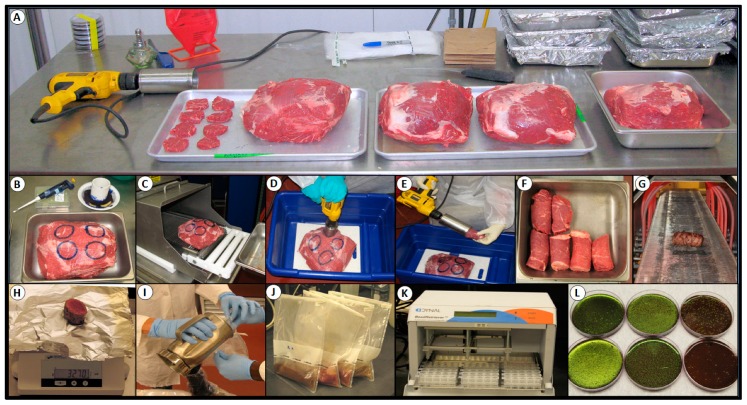
Phase 2, evaluation of select antimicrobials against *E. coli* O157:H7-inoculated beef subprimals in conjunction with blade tenderization. (**a**) Array of beef subprimals during one trial; (**b**) beef subprimal prepared for inoculation in labeled zones; (**c**) inoculated beef subprimal entering blade tenderizer; (**d**) sprayed and blade-tenderized subprimal being cored; (**e**) recovery of beef cores using a two-inch circular drill bit; (**f**) recovered beef cores; (**g**) sanitizing outside of beef cores by passage through radiant heat oven; (**h**) sectioning of beef cores; (**i**) blending and (**j**) enrichment incubation of beef sections; (**k**) Bead Retriever^TM^ for immunomagnetic bead recovery and enrichment of *E. coli* O157:H7 from enrichment broth; (**l**) plating of immunomagnetic beads with recovered *E. coli* O157:H7 onto EMB media containing rifamycin SV.

Top butt beef subprimals (IMPS #184) were acquired from a local wholesale distributor and “caps” were removed. The beef subprimals were processed using a coring device comprised of an electric hand drill fitted with a 2-inch diameter drill bit manufactured from a stainless steel pipe (Figure 2). The drill and bit were used to obtain beef cores that were used for making lean beef wafers (Figure 1 and Figure 2). Lean beef wafers were made by tempering beef cores for 1 h at −26 °C to firm them up, and then sliced on a Bizerba (Bizerba GmbH and Co. KG, Balingen, Germany) slicer to a thickness of 0.64 cm to create lean wafers of 5.1-cm (2-in) diameter (20.3 cm^2^); alternatively, lean beef wafers were obtained by slicing beef subprimals to desired thickness as large sheets on a slicer, and then manually coring individual wafers in a cookie-cutter fashion.

### 2.4. Antimicrobial Spray and Blade Tenderization System

A TC-700MC tenderizer (Ross Industries, Midland, VA, USA) equipped with a Dosatron (Clearwater, FL, USA) multi-nozzle custom-built spray system, with three nozzles spraying from above the conveyor belt, two from the sides, and five nozzles from below the belt (Figure 1) was used for the application of antimicrobial test solutions and non-lethal water control spray streams to the inoculated lean beef wafers (Phase 1) and beef subprimals (Phase 2). The two-blade tenderizing heads were removed from the Ross tenderizer when spray treating inoculated lean beef wafers, since no actual tenderization was taking place, and then replaced when spray intervention of inoculated beef subprimals was being performed in conjunction with blade tenderization. Each of the two blade heads consisted of seven rows that each contained 32 stainless steel blades (3 mm wide); the 14 rows of 32 blades contained in the two blade heads were positioned crosswise across the conveyor belt. The belt advanced in 3.6-cm increments with the blades penetrating the subprimals, after each advancement. Each subprimal was penetrated by both sets of blades during its 18 s dwell time, and it was determined that the system made 32 blade penetrations per 6.5 cm^2^ (~1 in^2^). The various antimicrobial and control treatment (water) solutions were held at 18–20 °C and sprayed at the maximum rate of 1.5 gallons (5.68 L) per minute with 40 pounds per in^2^ (psi) pressure. A restrictor valve was present that could reduce the spray rate by approximately half if needed.

### 2.5. Antimicrobial Solutions—Phase 1 (Lean Beef Wafers)

Fourteen antimicrobials were evaluated in this study at levels recommended by the manufacturer’s or distributor’s technical representatives: disodium metasilicate (AvGard-XP; Danisco A/S, Copenhagen, Denmark), cetylpyridinium chloride (Cecure; Safe Foods Corp., Little Rock, AR, USA), copper sulfate pentahydrate (Preserv; Envirogreen Global Solutions, Miami, FL, USA), sodium chlorite/citric acid/sodium hydroxide (Stabilized Na Chlorite; Alliance Analytical Laboritories Inc., Coopersville, MI, USA), peroxyacetic acid (Perasan; Enviro Tech Chemical Services Inc., Modesto, CA, USA), lauric arginate with peroxyacetic acid (CytoGuard Plus; A&B Ingredients, Fairfield, NJ, USA), acidified sodium chlorite (XG-940; Dan Mar Co., Arlington, TX, USA), sodium chlorite and citric acid (acidified sodium chlorite; Crimson Chemicals, Fort Worth, Tx, USA), lactic and citric acid (BeefXide; Birko Corporation, Henderson, CO, USA), hydroxypropanoic acid (Lactic Acid FCC 88%; Archer Daniels Midland Company, Decatur, IL, USA), hydrochloric and citric acid (Syntrx 3300; Synergy Technologies Inc., Shreveport, LA, USA), buffered sulfuric acid (AFTEC 3000; Advanced Food Technologies, LLC, Shreveport, LA, USA), hydrochloric and citric acid (Citrilow; Safe Foods Corp.), and hydrobromic acid (HB2; Enviro Tech Chemical Services, Modesto, CA, USA). The active ingredient concentrations were listed if available, otherwise the application strength of the diluted product solution that was received is given since some formulations were non-disclosed for proprietary purposes by respective manufacturers (Table 1). The pH for each particular antimicrobial used is also presented (Table 1). Antimicrobials showing maximum log reduction in Phase 1 (inoculated beef wafers) were selected for use in Phase 2 (whole muscle study).

### 2.6. Antimicrobial Solutions—Phase 2 (Blade Tenderization)

Seven of the 14 antimicrobials evaluated in Phase 1 of this study were further examined for a reduction in the translocation of viable *E. coli* O157:H7 in conjunction with the blade tenderization of beef subprimals. Antimicrobials showing maximum log reduction in Phase 1 (beef wafer study) through 7-days were selected for use in Phase 2 (whole muscle study). The antimicrobials used in Phase 2 were: AvGard-XP, Stabilized sodium chlorite, CytoGuard Plus, Lactic acid, AFTEC 3000, Citrilow, and, HB2 and were applied as recommended by the manufacturers (Table 1).

### 2.7. Inoculation and Spray Treatment of Lean Beef Wafers

Lean beef wafers were inoculated with 0.1 mL of ~2 × 10^8^ CFU/mL (i.e., ~1 × 10^6^ CFU/cm^2^) of a multi-strain *E. coli* O157:H7 cocktail while resting in sterile stainless steel trays (Figure 1 and Figure 2). After inoculation, the cocktail was spread over the surface of the wafers using a gloved finger. The tray was then covered with aluminum foil and allowed to sit at 3 °C for ~30 min to allow for bacterial attachment. The inoculated lean beef wafers were then subjected to an 18 s spray treatment for each antimicrobial product (*n* = 16 wafers × 4 post-treatment processing times = 64 wafers/antimicrobial).

Spray treatment was accommodated by running the samples through the Ross spray-integrated blade tenderizer. Although the TC700M tenderizer had a several gallon reservoir capacity, it was connected to an additional chemical reservoir in an adjoining room via connection through the wall that was outfitted with an automated sensor (Figure 1); as the level in the TC700M reservoir reached a low volume, the pump was triggered and the additional solution was pumped into the TC700M reservoir as needed. At the start of each trial, both reservoirs were filled to insure sufficient processing capacity for each antimicrobial trial. After each antimicrobial, the antimicrobial spray reservoir was allowed to run until it exhausted all of its contents, then rinsed thoroughly with hot water (70 °C) in order to ensure that residuals of prior antimicrobials and microbial inocula were eliminated from the chemical reservoir tank and conveyor belt chamber, respectively. Finally, the spray tank reservoir was filled twice, once with hot water and then again with deionized water and each was sprayed into the machine until depleted to remove the prior solution from the pump, adjoining lines, and tenderizer reservoir (approximately 2–3 min each).

Each spray trial run included three control groups: un-inoculated wafers (*n* = 8) subject to antimicrobial spray treatment, inoculated control wafers absent to spray treatment (*n* = 8), and inoculated wafers sprayed with deionized water (*n* = 8). Lean beef wafers subjected to these control treatments were processed within 1 h after spray treatment; controls were processed with each antimicrobial trial run. Four treatment group processing times were devised for the inoculated wafers sprayed with antimicrobials based on the time between spray treatment and final plating (~1 h, and 1-, 7-, and 14-days). Lean wafers were placed inoculated side up on the blade tenderizer conveyor belt and subjected to an 18 s dwell (spray) time. The 10 spray nozzles collectively expelled the particular antimicrobial or water control treatment at a rate of 5.68 L per minute. Wafers were then collected from the opposite end of the blade tenderizer machine and placed on absorbent pads in stainless steel trays, and then into sterile, filter-lined sampling stomacher bags (Nasco Whirl-Pak^®^). The same process was conducted for the water treatment and un-inoculated controls subjected to antimicrobial spray treatment. Samples retrieved from the blade tenderizer were then placed into sterile stomacher filter bags (Nasco; Figure 1). Microbial enumeration of lean beef wafers was obtained by placing two beef wafers per sterile filter membrane sampling bag and adding 40.6 mL of Dey-Engley Neutralization Media (D/E broth, Becton-Dickinson) prior to stomaching in a paddle blender at a high speed for 60 s; subsequent dilutions were then made in 0.1% buffered peptone water (BPW, Difco). One portion of wafers were processed immediately (i.e., ~1-h, *n* = 16) and the remaining sets of wafers were vacuum-sealed, stored at 3 °C, and processed for the *E. coli* O157:H7 inoculum counts after 1- (*n* = 16), 7- (*n* = 16), and 14-days (*n* = 16). Data obtained for these samples are provided in a Supplemental Data File.

### 2.8. Inoculation and Mechanical Tenderization of Beef Subprimals

Top sirloin butt beef subprimals (IMPS #169; ca. 10–15 lb each) were purchased fresh from a local wholesale distributor and stored at 4 °C for no longer than seven days prior to use. The caps were removed and fat was trimmed so that a contiguous intact core could be obtained (Figure 2). For each tenderization treatment, four 5.1-cm diameter circles were marked using edible ink on the lean surface of the beef subprimals (Figure 2). One hundred microliters of a multi-strain cocktail of *E. coli* O157:H7 (~2 × 10^6^ CFU/mL) was applied to each circle and then thoroughly spread with a gloved finger within the circles (each ~20.3 cm^2^; i.e., ~1 × 10^4^ CFU/cm^2^). The inoculated top sirloin butt beef subprimal was then allowed to sit at 4 °C for 30 min to promote the attachment of the cells to the meat surface prior to any further processing. Each of the subprimals were moved via a conveyor belt (inoculated side up) on the TC 7000M tenderizer (Ross Industries; Figure 2) equipped with a custom-built multi-nozzle spray system (Dosatron) that eventually led the meat beneath two sets of tenderizing blades in tandem. The integrated spray intervention system was comprised of three nozzles spraying from above, two nozzles spraying from the sides, and five spraying from below the conveyor belt. For blade tenderization with beef subprimals, there were two control treatments per trial: inoculated with water spray and blade tenderization (W+BT) that served as the positive translocation control, and inoculated with water spray but without blade tenderization (W−BT) that served as the negative translocation control. Treatments consisted of inoculated subprimals subjected to antimicrobial spray treatment and blade tenderization (A+BT). Each treatment passed once longitudinally through the tenderizer, lean side (inoculated side) up with a water spray. A top sirloin butt beef subprimal was set up with four separated replicates of inoculated circles (20.3-cm^2^) per top sirloin butt for each antimicrobial. The antimicrobials were then processed at a single concentration, as recommended by the manufacturer’s technical personnel.

### 2.9. Microbial Sampling, Isolation, and Confirmation

For enumeration of inoculated *E. coli* O157:H7 from lean beef discs in our Phase 1 trials, two discs were placed in each stomacher bag (Figure 1; the inoculated side was placed facing the filter membrane which could serve as an abrasive rub during stomaching) to which 40.6 mL of Difco^TM^ D/E Neutralizing Broth (Becton-Dickinson) was added and stomached at the “high” setting for 60 s with a Model 400 stomacher (Seward Laboratory Systems Inc., Bohemia, NY, USA). Additional dilutions were made and samples were surface-plated onto Bacto^TM^ Tryptic Soy Agar (TSA) (Becton-Dickinson) containing gentamycin and rifamycin SV, as mentioned earlier. The plates were incubated at 37 °C for 48 h, at which time the plates were counted manually for antibiotic-resistant (*E. coli* O157:H7) colonies. This same protocol was followed for treatment groups processed following 1-, 7-, and 14-days of refrigerated storage. Un-inoculated control lean beef disc samples were also plated to insure the absence of counts from the meat source.

Microbial evaluation of meat core samples obtained in Phase 2 after spray intervention and blade tenderization were processed for the presence/absence of our Gen^R^/Rif^R^ *E. coli* O157:H7 inoculum using the regimen depicted in Figure 2. After removing the beef cores from the beef subprimals, approximately 1/8-in of the inoculated surface was removed and the needle-entry side (top) was marked with edible ink. The beef cores were then surface-sanitized from *E. coli* O157:H7 contamination that might have been acquired during liquid spray dispersal and subsequent core removal by running them through a radiant heat oven (IR Grill^Tm^, Marlen/Unitherm Food Systems, Bristow, OK, USA) for five min at high heat (~approx. 500 °F air temperature at the beef surface; Figure 2). Upon exiting the radiant oven, the beef cores were aseptically sectioned into 1-inch sections that were weighed (~45–50-g), placed into a stainless steel Waring blender with 100 mL enrichment broth (modified TSB supplemented with novobiocin, 10 µg/mL), and blended at “high” speed for 1 min. Blended samples were then poured into sterile filter-lined stomacher sampling bags and incubated at 37 °C. After 24 h enrichment, each sample bag was stomached to mix the contents and 1 mL was removed for extraction with *E. coli* O157:H7-specific immunomagnetic beads (Invitrogen, ThermoFisher Scientific, Waltham, MA, USA) using the USDA-FSIS method for immunomagnetic separation (IMS) recovery on an automated Bead Retriever^Tm^ (Dynal, ThermoFisher Scientific; Figure 2). After recovery by the IMS procedure, the entire magnetic bead-cell mixture was plated onto Levine’s eosin methylene blue agar (EMB, Oxoid, Cambridge, UK) containing rifamycin SV. The plates were read after 24 h of incubation at 37 °C as positive (those with the characteristic *E. coli* metallic sheen) or negative for *E. coli* O157:H7 (if green sheen colonies were absent) (Figure 2).

### 2.10. Statistical Analysis

For each set of treatments, duplicate platings were made at each dilution level for each set of combined lean beef disc samples at each time tested and averages were calculated. The surface counts of *E. coli* O157:H7 from lean beef wafers were transformed into log CFU per square cm form (CFU/cm^2^). Standard deviation of the log CFU/cm^2^ values associated with each antimicrobial were calculated using the statistical function option offered with Microsoft Excel 2016 software (Redmond, WA, USA) for Phase 1 and the STDEVPA function for Phase 2. Log reduction values were considered dependent variables. Data were analyzed using version 13.0 of the Sigma Plot statistical package (Systat Software Inc., San Jose, CA, USA). A one-way analysis of variance (ANOVA) was performed and pairwise multiple comparison procedures (Holm-Sidak method) were used for mean separation of log reduction values; differences were considered significant at *p* values of <0.05.

## 3. Results

### 3.1. Phase 1: Evaluation of 14 Antimicrobials against E. coli O157:H7 on the Surface of Inoculated Lean Beef Discs

**Figure 3:**
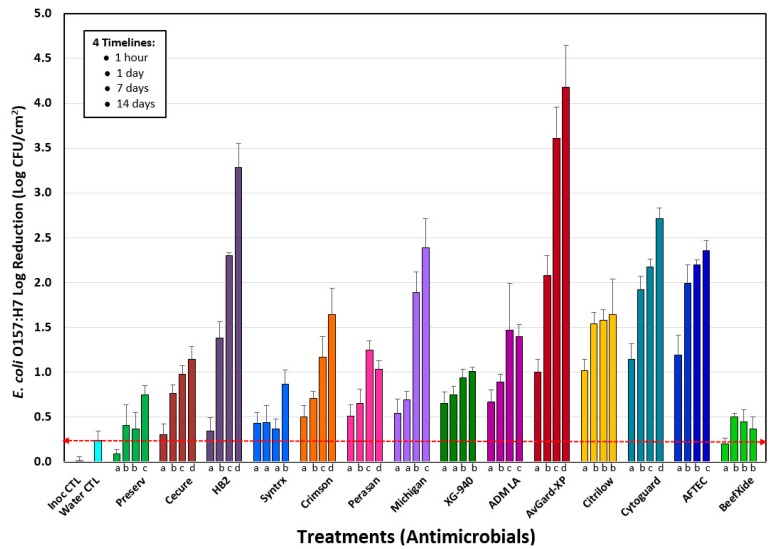
Reduction of *E. coli* O157:H7 on inoculated lean beef wafers passing through the tenderizer spray system. Data shows reductions observed relative to inoculated samples (Inoc CTL), water spray treatment (Water CTL), and 14 different antimicrobials. Groups of four data bars each represent samples processed within 1 h, 1-day, 7-days, and 14-days after treatment. Each antimicrobial data bar represents the mean of eight pairs of lean beef wafers (*n* = 16) and error bars represent the standard deviation of the means. The “inoculation control (Inoc CTL)” and “water control (Water CTL)” data bars represent the difference from the mean of four pairs of lean beef wafers (*n* = 8) conducted for each of the 14 antimicrobial trials (*n* = 8 × 14 = 112 wafers) and the standard deviation represents the variance within the enumerated counts of these controls. A red dotted line indicates the mean of the reduction obtained by non-lethal water displacement of inoculum (Water CTL). One-way analysis of variance (ANOVA) was applied to data within each group of four time points for each antimicrobial. Data bars with the same lowercase letter (below the bars) are not significantly different; those with different letters are significantly different (*p* < 0.05).

Phase 1 consisted of testing antimicrobials at supplier-recommended levels on *E. coli* O157:H7-inoculated lean beef discs to mimic *E. coli* on the surface of beef subprimals sprayed with antimicrobials. Processed beef disc samples were held on ice and processed within 1 h of treatment and additional sets of samples were vacuum-packaged and processed after 1-, 7-, and 14-days. Microbial counts obtained showed significant differences of *E. coli* O157:H7 counts by antimicrobials when tested within 1 h after processing vs. sampling after 1-, 7-, or 14-days (Figure 3). Untreated inoculated lean beef wafers served as primary controls to indicate the standard deviation within the base inoculation level (i.e., inoculum control, Inoc CTL) while others sprayed with water served as secondary controls to indicate the level of pathogen displaced by spray pressure (i.e., Water CTL) for comparison with samples spray treated with antimicrobials (Figure 3). No counts were obtained, or they were below the limit of detection, from un-inoculated beef wafers, sprayed with antimicrobials and enumerated on antibiotic media for which our *E. coli* O157:H7 inoculum was resistant to (Supplementary Data File).

Microbial surface counts of lean beef wafers processed within 1 h post-treatment with various antimicrobials revealed that at the concentrations used, AFTEC 3000, CytoGuard Plus, Citrilow, and AvGard-XP were among the most effective antimicrobials with surface reductions of 1.0–1.2 log CFU/cm^2^ within 1 h of treatment. The 1 h timeline reflects what a processor might expect if antimicrobials were used to reduce surface STEC some time shortly prior to mechanical tenderization. Actually, all the samples for the 1 h timeline were mixed with a D/E Neutralization Buffer immediately after processing through the spray system, but were further processed for plating within a 1 h timeframe; samples for the 1-, 7-, and 14-day timelines were vacuum packaged, refrigerated, and not mixed with D/E Neutralizing Buffer until just prior to microbial enumeration. These solutions proved more effective (*p* < 0.05) than the remaining tested antimicrobials at this time period (Figure 3).

The maximum level of reduction of *E. coli* O157:H7 by various antimicrobials differed depending on the post-process hold time before microbial enumeration. This was done to evaluate the potential reduction obtained at different hold times after treatment, whereby a supplier might spray an intervention, package the beef, and ship it to an end-user that would then perform blade tenderization at a later time. After the spray treatment and 1 day of refrigerated storage, AvGard-XP, AFTEC 3000, and CytoGuard Plus were the most effective (*p* < 0.05) at reducing surface counts (~2.1–1.9 log CFU/cm^2^) of *E. coli* O157:H7 (Figure 3). After seven days of refrigerated storage microbial enumeration showed that AvGard-XP was again the most effective (*p* < 0.05) antimicrobial tested (Figure 3) for the reduction of *E. coli* O157:H7 (~3.61 log CFU/cm^2^ log reduction) on the surface of lean beef wafers, followed by HB2, AFTEC 3000, CytoGuard Plus, and Stabilized Sodium Chlorite (~2.3–1.9 log CFU/cm^2^ reduction). Similarly, after 14 days of refrigerated storage, surface counts of lean been wafers revealed that AvGard-XP remained the most effective (*p* < 0.05) in the reduction (~4.18 log CFU/cm^2^) of *E. coli* O157:H7 surface load (Figure 3). HB2 was the second most effective (*p* < 0.05) antimicrobial used for surface reduction (~3.28 log CFU/cm^2^) of *E. coli* O157:H7 after 14 days post-process storage followed by CytoGuard Plus, Stabilized Sodium Chlorite, and AFTEC 3000.

### 3.2. Phase 2: Translocation of E. coli O157:H7 into Beef Cores

**Figure 4:**
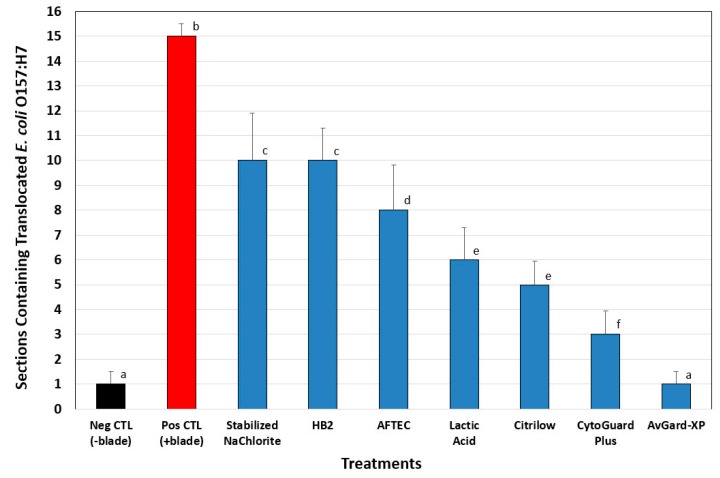
Reduction of *E. coli* O157:H7 in antimicrobial spray-treated, blade-tenderized beef as determined from individual core sections recovered after processing (i.e., inoculation, spray treatment, blade tenderization, core excised, sectioning, blending, enrichment, and microbial plating). Results for each treatment (four cores/treatment; four sections/core; *n* = 16 sections) were determined for the presence/absence of our inoculated *E. coli* 157:H7 strains. Negative controls represented inoculated but non-tenderized beef (Neg CTL), while positive controls represented inoculated and tenderized beef (Pos CTL). Positive and negative controls were sprayed with water instead of antimicrobials. Error bars represent standard deviation of *E. coli* O157:H7 presence (versus absence) within each set of the four replicate core samples, while bar height is the total of positive sections among four replications. Pairwise analysis of variance (Holm-Sidak method) of treatments with different letters represents significant differences (*p* < 0.05); data bars with the same letters are not significantly different.

Seven antimicrobials showing the largest log reductions in the beef wafer trials (Lactic acid ADM, CytoGuard Plus, AvGard-XP, Citrilow, AFTEC 3000, HB2, and Stabilized sodium chlorite) were chosen to be tested in conjunction with blade tenderization to determine the incidence of *E. coli* O157:H7 translocation into beef core sections. The antimicrobials chosen were based on results obtained by performance against *E. coli* O157:H7 through seven days post-treatment with inoculated lean beef discs. Inoculated beef subprimals that were sprayed with water but not blade tenderized (negative control) resulted in 1 of 16 sections (compilation of four beef core replications of four sections each) positive for *E. coli* O157:H7 whereas, inoculated beef subprimals sprayed with water and blade tenderized (positive control), resulted in 15 of 16 sections positive for *E. coli* O157:H7 (Figure 4). When inoculated beef subprimals were sprayed with antimicrobials and subjected to blade tenderization, we obtained various levels of translocation into various beef sections, all at levels lower than the positive controls (Figure 4). AvGard-XP was the most effective (*p* < 0.05) at reducing the translocation of *E. coli* O157:H7 into internal beef core sections, resulting in only one positive section for *E. coli* O157:H7 (Figure 4). CytoGuard Plus (+PAA), Citrilow, and Lactic Acid were the next effective group of antimicrobials at reducing translocation, resulting in 3–6 sections positive for *E. coli* O157:H7. Although the remaining solutions, HB2, AFTEC 3000, Stabilized Na-Chlorite were less effective (*p* < 0.05), they were still more effective relative to the positive controls using water alone (Figure 4).

## 4. Discussion

One of the initial works to suggest that mechanical tenderization may lead to food safety concerns was a graduate thesis from Kansas State University in 1996 (i.e., S. Sporing) and published several years later [13]. That work suggested possible concerns from the internalization of *E. coli* O157:H7 during the mechanical tenderization of beef steaks. Since those initial concerns, investigators have examined the possibility that the translocation of *E. coli* O157:H7 from the surface of beef subprimals to their interior during mechanical tenderization could be a cause for concern. The concern over STEC bacterial contamination of beef surfaces has prompted the application of numerous antimicrobial “decontamination” treatments on the surfaces of intact meats, from carcasses to small cuts, and even beef “trim” to eliminate or reduce their occurrence. All chemicals used on/in meat and poultry products (in the USA) must be approved first by the US Food & Drug Administration, which regulates all food ingredients for safety, and second by the USDA-FSIS, which regulates ingredients used in meat and poultry products on the basis of efficacy for its intended purpose. Antimicrobials allowed by USDA-FSIS on meat and poultry products may be found online in the “Safe and Suitable Ingredients List”, which is updated one or more times per year and includes maximum use levels [22]. When used for “antimicrobial” purposes, the efficacy that must be demonstrated is the microbial reduction of the targeted organism of concern. Many antimicrobials are inorganic or organic acids, or combinations of them. Inorganic acids provide a low pH toxic environment that is biocidal to many bacteria. Organic acids (acetic, citric, lactic), in addition to low pH, are inhibitory to bacteria when they are taken up by bacterial cells usually when the pH is below their acid dissociation constant (pK_a_), so that the organic acid molecule is more likely to be associated and chemically neutral; once inside bacterial cells, the pH (~pH 7.0) is above the pK_a_ of most organic acids and they then dissociate whereby the anion form of the acid becomes internally toxic to the cell [23,24]. Peroxyacetic acid and other oxidants are biocidal by denaturing proteins, oxidizing sulfhydral bonds in proteins and enzymes, and are generally disruptive to the integrity of the cell wall [25]. The mode of action underlying the effects of copper sulfate is not well understood although embedded copper is known to have “contact” inhibition of various microbes by interaction with the bacterial membrane [26]. The use of copper sulfate allows a more solubilized form to be administered [27]. Acidified sodium chlorite is produced by the addition of organic or inorganic acids to sodium chlorite, and the antimicrobial effect is considered due to its oxidative properties. Cetylpyridinium chloride is a cationic quaternary ammonium compound (QAC), which is generally known to cause cell leakage through membrane damage. They are commonly used in mouthwash rinses and have been increasingly examined for applications in meat and poultry. Disodium metasilicate is a highly basic and corrosive compound that affects bactericidal activity likely due to its high pH, and has been suggested to also disrupt cell membranes causing leakage and loss of cell integrity [28,29].

The use of “lean beef discs” as a surface spray model substrate facilitated the screening of numerous antimicrobials and samples. Prior to spray treatment, a ~30-min hold period was used to facilitate the bacterial attachment to lean beef discs after inoculation. This period was sufficient, as the inoculum held tenaciously to the inoculated meat surface in control treatments when sprayed with water, resulting in only a 0.24-log (±0.10-log) reduction. This number was not subtracted from the antimicrobial treatments to indicate net lethality due to antimicrobials. After treatment, we applied a diluent volume (20.3 mL) equivalent to each beef disc inoculated surface area (20.3 cm^2^) that would allow enumeration of “per mL” solution that would be equivalent to the inoculated surface area on a “per cm^2^” basis; all microbial levels could thereby be related to each other and to the treatment received. Also, the beef disc surfaces were positioned in the filter membrane bags such that the inoculated surfaces faced the membrane to receive an abrasive treatment during stomaching as opposed to facing the smooth plastic bag surface.

The difference observed with some antimicrobials between 1 h to 1-day sampling result brings up a concern we have had with “overnight shipment” of test samples to an outside/distant commercial testing lab, as is often done by industry without in-house testing facilities or for large projects where same-day enumeration is not feasible. For instance, Cecure, AvGard-XP, Citrilow, CytoGuard, and AFTEC 3000 showed significantly lower results (*p* < 0.05) when enumerated at 1 h (0.45-, 1.06-, 1.03-, 1.16-, 1-18-log reduction, respectively) after processing compared to enumeration the next day (0.91-, 2.07-, 1.54-, 1.95-, 1.98-log reduction, respectively). For these samples, if one had to depend on data provided by next day testing, the conclusion might be that the spray intervention provided the 24 h result immediately upon application of the antimicrobial. The difference might be the result of adding D/E Neutralizing Broth immediately to the 1 h samples (as a diluent) prior to stomaching/enumeration, while this wasn’t done to the later samples until just before they were processed. This suggests that care should be taken on what kind of samples should be submitted for overnight shipment, versus having them sampled as soon as possible. In some cases, it might be desirable to delay microbial enumeration to incur a greater impact of residual antimicrobial (i.e., commercial benefit) while in other cases it may not.

Another aspect that our timed study brought out (Figure 3) was the potential issue of “processing aid vs. ingredient”. According to federal labeling requirements, processing aids are defined as, “substances that are added to a food for their technical or functional effect in the processing but are present in the finished food at insignificant levels and do not have any technical or functional effect in that food” [30,31], and therefore, are not required to be labeled. This brings into consideration whether the antimicrobial has residual activity, whereby microbial reduction continues to occur over time as observed in Figure 3. Although these are specifics to be argued at the point of process implementation, our studies were not intended to be an exhaustive treatise on antimicrobial spray treatment, as we examined a considerable number of potential antimicrobials in one-off trials based on levels recommended by various suppliers. It should be noted that the effectiveness of each of these antimicrobials could be further optimized or improved relative to the results observed herein by additional studies. Our data confirms those observed by Heller et al. [32] in which antimicrobial spray interventions prior to mechanical tenderization could reduce surface viability and therefore the incidence of internal translocation of surface STEC.

Due to the various potential sizes of meat cuts, from steaks to large subprimals, we applied our inoculum according to a standardized bacterial surface density (CFU/cm^2^). We believe it is the bacterial surface density that determines the likelihood of a (clean) tenderizing blade to come in contact with bacterial cells and draw them into the interior; a higher surface population density would be more likely to contribute to this while a lower cell density would less likely contribute (i.e., at a lower rate). Similarly, loss of viability due to antimicrobial spray intervention changes the viable population to a somewhat lower (viable) cell density, reducing the likelihood of needle translocation. Standardizing the inoculated population density on the surface of different-sized beef cuts also standardizes the population that would be effected by antimicrobials, since it is at the surface where the antimicrobial spray addresses the surface microbiota. If an inoculum was applied on a weight basis of the beef products tested, then one could experience significantly different bacterial surface population densities if small versus large beef products were being treated. This could change the risk of needle-based surface entry into beef between samples and/or the impact of different antimicrobials being applied to different population densities on different beef samples. Although our beef was procured fresh and of excellent quality, we are unaware of any published studies demonstrating that different physical conditions of beef (moisture, temperature) play a role in bacterial translocation efficiency, as they certainly may. Our data shows that antimicrobials demonstrating good antimicrobial performance in the lean beef wafer assays also performed well (i.e., fewer positive sections) in reducing the number of positive sections obtained after blade tenderization (Figure 4) compared to positive controls with no antimicrobial treatment. The degree of surface reduction is reflected in the reduction of translocation frequency as seen in Figure 3 and Figure 4.

We examined sections of beef cores measured in 1-inch sections from the point of blade entry during mechanical tenderization, keeping track of sections in the order in which they occurred. It was observed that when antimicrobials were applied, the number of sections that were STEC-positive started to decrease, relative to the positive control. Those sections that were farthest from the needle entry side were mostly negative compared to those close to the needle entry side. We consider this an indication that the level of *E. coli* O157:H7 was diminished on the surface that translocation by blade entry was only sufficient to inoculate the first 1-to-3 sections of the 4 sections, whereas the higher inoculation level (i.e., 10^4^ CFU/cm^2^) of the positive control allowed entry all the way to the fourth section. We determined in preliminary trials that 10^4^ CFU/cm^2^ allowed us to discern differences between the antimicrobial and controls that were not observed at 10^6^ CFU/cm^2^ (all sections positive) nor at 10^2^ CFU/cm^2^ (too few sections positive). Although 10^4^ CFU/cm^2^ far exceeds the level likely to be found on any commercial beef product, such levels are necessary to define differences in translocation after treatment with the various antimicrobials that would otherwise not be detected if inoculated at actual retail beef contamination levels.

The USDA-FSIS has referred to such mechanically tenderized products as non-intact beef and indicated that processors and end-users should re-evaluate the processes that involve this technology for possible safety concerns [12]. USDA-FSIS proposed a rule (2013) that mechanically-tenderized beef should be labeled, as such so that potential customers may be informed as to the potential risk from consuming such products and a final rule has recently been issued and enacted as of May, 2016 [16]. This issue was hard fought between consumer interest groups claiming that consumers have a “right to know” if there is a risk from consumption of mechanically tenderized beef, versus. industry groups suggesting that labeling would hurt their industry. USDA-FSIS was caught in the middle of this argument. During our study, we used a radiant oven (InfraRed Grill, Figure 2) to “sanitize” the outside of our recovered beef cores from potential surface contamination. This process of sanitizing the outside of the recovered beef cores to insure that recovered STEC was from internalized cells also simulated the exact consumer issue. That is, by cooking beef at high heat on the outside while still being rare on the inside, it still allowed the recovery of *E. coli* O157:H7 that was translocated to the interior of the meat by mechanical tenderization. One food safety approach is to assure that all steaks are cooked to a safe endpoint insuring no survival of blade-translocated organisms. Studies have examined heat lethality of various cooking methods [19,20] or the thickness of steak cuts [33,34]. However, not everyone enjoys medium-to-well-cooked steaks. Recent data show that heat-resistant STEC possessing the locus of heat resistance (LHR) are still able to survive at >9+ log difference over non-heat-resistant strains when heated at 60 °C for 15 min [35].

## 5. Conclusions

Our data show that the implementation of antimicrobial spray interventions can result in a reduced risk of translocated *E. coli* O157:H7 to interior sections. It is possible that effective industry-based interventions could lead to softening the meat labeling requirements for mechanically tenderized products. A similar “loosening” of USDA-FSIS testing for *Listeria monocytogenes* is currently accommodated in the ready-to-eat (RTE) processed meat industry by defining product processing categories based on risk (i.e., Alternative 1, 2, or 3) according to the final rule for control of *L. monocytogenes* in RTE meats [36]. If processors incorporate antimicrobial interventions as either a post-process lethality step or antimicrobial ingredients to prevent the growth of *L. monocytogenes* during shelf life (or both), the products are considered by USDA-FSIS as having a “reduced risk” and rewarded by being tested less than products not receiving the interventions. The same reduced-risk scenario could be incentivized for mechanically tenderized beef whereby those that receive a validated antimicrobial intervention reduce the presence of potential surface pathogens, and therefore reduce the risk of internalization (internal translocation). It could possibly have some type of incentive awarded, such as a waiver of the labeling requirement. Of course, similar to the interventions implemented for control of *L. monocytogenes* on RTE meats, they would be required to be validated with appropriate studies by qualified individuals. The application of antimicrobial surface interventions to address surface microbiota before mechanical tenderization may provide the best approach to facilitate reduction during heating, which is often an issue of both heating time and microbial load.

## Figures and Tables

**Table 1:** List of antimicrobials used in Phase 1 (inoculated lean beef wafers).

Product/Trade Name		Active Ingredient(s)	pH	Application Strength
1.	AvGard-XP	Disodium metasilicate	13.1	60,000 ppm SMS
2.	HB2	Hydrobromic acid	7.5	300 ppm
3.	Cecure	Cetylpyridinium chloride	7.0	4000 ppm
4.	Preserv	Copper sulfate pentahydrate	6.8	30% of concentrate *
5.	Stabilized Sodium Chlorite	Sodium chlorite, citric acid, sodium hydroxide	6.5	<1%, <1%, <1% each *
6.	XG-940	Acidified sodium chlorite	6.5	200 ppm
7.	Perasan MP2	Peroxyacetic acid	3.2	220 ppm
8.	CytoGuard Plus	Lauric arginate (LAE), peroxyacetic acid (PAA)	3.0	5,000 ppm LAE; 220 ppm PAA
9.	Acidified Sodium Chlorite	Sodium chlorite acidified with citric acid	2.7	1100 ppm
10.	BeefXide	Lactic acid, citric acid	2.1	2.4% of concentrate *
11.	Lactic Acid	Hydroxypropanoic acid	1.9	50,000 ppm
12.	Syntrx 3300	HCl, citric acid	1.2	3% of concentrate *
13.	AFTEC 3000	Buffered sulfuric acid	1.0	17,500 ppm
14.	Citrilow	HCl, citric acid	0.8	18% of concentrate *

* For proprietary reasons the actual concentrations of some solutions have not been disclosed; the “application strength” listed is the dilution level of the concentrate provided by the manufacturer or approximate level of active.

## Supplementary Materials

The supplementary data is available online at http://www.mdpi.com/2304-8158/8/2/80/s1.
